# Receptors for Respiratory Syncytial Virus Infection and Host Factors Regulating the Life Cycle of Respiratory Syncytial Virus

**DOI:** 10.3389/fcimb.2022.858629

**Published:** 2022-02-25

**Authors:** Ziheng Feng, Lili Xu, Zhengde Xie

**Affiliations:** ^1^Beijing Key Laboratory of Pediatric Respiratory Infection Diseases, Key Laboratory of Major Diseases in Children, Ministry of Education, National Clinical Research Center for Respiratory Diseases, National Key Discipline of Pediatrics (Capital Medical University), Beijing Pediatric Research Institute, Beijing Children’s Hospital, Capital Medical University, National Center for Children’s Health, Beijing, China; ^2^Research Unit of Critical Infection in Children, Chinese Academy of Medical Sciences, Beijing, China

**Keywords:** respiratory syncytial virus, receptors, host factors, infection, virus–host interaction

## Abstract

Respiratory syncytial virus (RSV) is a common cause of lower respiratory tract infections and responsible for a large proportion of mortality in children and the elderly. There are no licensed vaccines available to date. Prophylaxis and therapeutic RSV-specific antibodies are limited to populations at high risk owing to high cost and uncertain clinical value. Receptors and host factors are two determinants important for virus entry and establishment of infection *in vivo*. The identification and understanding of viral receptors and host factors can help us to gain insight into the pathogenesis of RSV infection. Herein, we reviewed receptors and host factors that have been reported thus far. RSV could bind to CX3C chemokine receptor 1 and heparan sulfate proteoglycans *via* the G protein, and to nucleolin, insulin-like growth factor-1 receptor, epidermal growth factor, and intercellular adhesion molecule-1 *via* the F protein. Seven host restriction factors and 13 host factors essential for RSV infection were reviewed. We characterized the functions and their roles in the life cycle of RSV, trying to provide an update on the information of RSV-related receptors and host factors.

## Introduction

Respiratory syncytial virus (RSV) is one of the most common viral etiology of lower respiratory tract infections and a major cause of mortality in children younger than 5 years old and the elderly worldwide, imposing a huge disease burden on public healthcare system (Scheltema et al., 2017; Geoghegan et al., 2017). To date, there are no available licensed RSV vaccines and specific antiviral therapies for RSV infection. Passive immunization using RSV-specific monoclonal antibodies, such as palivizumab, is of high cost, and the value for prophylaxis in populations at high risk of severe infection remains unclear (Mac et al., 2019). Looking for underlying RSV therapeutic targets and developing alternative RSV-specific prophylaxis and therapies are needed.

RSV is a filamentous enveloped, non-segmented, negative-sense, single-stranded RNA virus, belonging to the *Orthopeumovirus* genus of the Pneumoviridae family in the Mononegavirales order (Battles and McLellan, 2019). Externally transmembrane glycoproteins (glycoprotein [G], fusion protein [F], small hydrophobic protein [SH]) are crucial for RSV attachment and fusion (Griffiths et al., 2017). Several receptors have been described for RSV entry and pathogenesis (**Table 1**), including CX3C chemokine receptor 1 (CX3CR1) (Tripp et al., 2001), nucleolin (Tayyari et al., 2011), epidermal growth factor (EGFR) (Currier et al., 2016), insulin-like growth factor-1 receptor (IGF1R) (Griffiths et al., 2020), heparan sulfate proteoglycans (HSPGs) (Feldman et al., 1999), and intercellular adhesion molecule-1 (ICAM-1) (Behera et al., 2001). While RSV G protein binds to CX3CR1 and HSPG, RSV F protein can interact with nucleolin, EGFR, IGF1R, and ICAM-1. Among these receptors, CX3CR1 is the most likely candidate because its expression pattern matches RSV tropism and the interaction between CX3CR1 and CX3C motif within RSV G protein contributes to suppression of interferon type I (IFN-I) and lead to Th2-polarized response, which are corelating to what have been observed in many studies (Isaacs, 1989; Pinto et al., 2006; Cormier et al., 2014; Caballero et al., 2015; Hijano et al., 2019), although other viral proteins also participate in the processes. HSPGs are the primary receptors for RSV entry into immortal cell lines *in vitro*, but it may not be necessary receptors in the human epithelium *in vivo* given their basal expression in these cells. The receptors binding to the F protein mainly facilitate host cell infection by mediating attachment, fusion, and macropinocytosis. There is some overlap in terms of functions between the G protein and F protein.

**Table 1:** Receptors for RSV infection.

Receptors	Binding to	Functions	References
CX3CR1	G protein	mediating virus attachment; inhibiting IFN type I production; promoting Th2-polarized response.	(Imai et al., 1997; Tripp et al., 2001; Harcourt et al., 2006; Chirkova et al., 2013; Chirkova et al., 2015; Jeong et al., 2015; Johnson et al., 2015; Zhivaki et al., 2017; Anderson et al., 2020; Green et al., 2021)
Nucleolin	F protein	mediating virus internalization	(Tayyari et al., 2011; Hegele, 2012; Griffiths et al., 2020; Mastrangelo et al., 2021)
EGFR	F protein	inducing macropinocytosis of RSV; promoting virus fusion; increasing airway mucus secretion.	(Currier et al., 2016; Lingemann et al., 2019)
IGF1R	F protein	facilitating the translocation of nucleolin to cell membrane	(Griffiths et al., 2020)
HSPGs	G protein	virus attachment.	(Krusat and Streckert, 1997; Bourgeois et al., 1998; Feldman et al., 1999; Hallak et al., 2000; Feldman et al., 2000)
ICAM-1	F protein	facilitating neutrophils and eosinophils adhesion to airway	(Patel et al., 1995; Arnold and Konig, 1996; Chini et al., 1998; Wang et al., 2000; Behera et al., 2001)

The establishment of virus infections in the host is greatly affected by host–virus interactions. Host restriction factors are cellular proteins constituting the first line of defense against viruses. They exhibit enormous diversity in structures and functions. Host restriction factors can restrict viral infection by targeting and interfering with almost every step of the viral life cycle. In addition, host restriction factors act as potent barriers against cross-species transmission. Host restriction factors have several characteristics: (1) most of them are germ-line encoded, cell-intrinsic proteins expressed in almost all cell types; (2) in addition to their generally IFN-inducible feature, many host restriction factors are constitutively expressed in different cell types, enabling their rapid responses against viruses; (3) they frequently target conserved viral components; and (4) they have biological functions outside of immunity, and some of the restriction effects might be attributed to their cell-regulatory functions (Kluge et al., 2015). To date, there are only a few host restriction factors that have been identified to inhibit RSV infection in humans (**Table 2**), including guanylate binding protein 5 (GBP5) (Li et al., 2020), chemokine ligand 4 (CXCL4) (Han et al., 2020), extraribosomal L13a (Mazumder et al., 2014), interferon-induced transmembrane proteins (IFITM) (Zhang et al., 2015), apolipoprotein B mRNA-editing enzyme-catalytic polypeptide 3G (APOBEC3G; A3G) (Fehrholz et al., 2012), and interferon-induced protein 44 and interferon-induced protein 44-like (IFI44 and IFI44L) (Busse et al., 2020).

**Table 2:** Host factors for RSV infection.

Host factors	Functions	References
***Host restriction factors***		
GBP5	promoting SH protein secretion from cells	(Li et al., 2020)
CXCL4	blocking RSV binding to HSPGs	(Han et al., 2020)
L13a	promoting formation of VAIT complex and silencing M proteins translation	(Mazumder et al., 2014)
IFITM	interfering with viral entry and replication; reducing cellular infiltration in lung	(Everitt et al., 2013; Zhang et al., 2015; Smith et al., 2019)
IFI44 and IFI44L	restricting viral replication and transcription	(Busse et al., 2020)
A3G	impaired viral transcription; inhibited replication; enhanced mutation rate	(Fehrholz et al., 2012)
***Host factors essential for RSV infection***		
TLR4	increasing neutrophils infiltration in lung	(Kurt-Jones et al., 2000; Monick et al., 2003; Puthothu et al., 2006; Goutaki et al., 2014; Zhou et al., 2016)
CD14	having synergistic effect with TLR4 on the pathogenesis	(Kurt-Jones et al., 2000; Puthothu et al., 2006; Goutaki et al., 2014)
ATP1A1	inducing macropinocytosis of RSV by activating EGFR	(Lingemann et al., 2019)
ARP2	mediating budding and virus production; involving in the processes of F protein induced filopodia formation	(Mehedi et al., 2016)
ABCE1	promoting virus replication and progeny virus assembly	(Anderson et al., 2019)
Rab5a	facilitating the formation of macropinosomes; inhibiting IFN-g production	(Mo et al., 2021)
JNKs	suppressing the expression of TNF-α; associating with RSV assembly and progeny virion release	(Caly et al., 2017)
Importin β1	importing M protein into nucleus	(Ghildyal et al., 2005)
Cof1	increasing F actin filaments; promoting RSV proteins trafficking and virus transcription in inclusion bodies	(Kipper et al., 2015)
Cav2	maintaining lipid raft integrity essential for stability of viral filaments; facilitating the progeny viruses release	(Kipper et al., 2015)
ZNF502	unclear	(Kipper et al., 2015)
Crm1	inducing M protein shuttling from nucleus to cytoplasm	(Ghildyal et al., 2009)
p32	Unclear	(Hu et al., 2017)

In the long, continuous virus–host arm race, viruses have evolved a series of adaptative mechanisms to not only avoid their natural host immunity and restriction but also enable viruses to utilize some cellular proteins to promote their replication (Doyle et al., 2015). The definition of the host factors essential for virus replication is ubiquitous. The mechanism of their facilitating properties in virus replication is poorly characterized, and most of their functions have been demonstrated by loss-of-function screens. Only several host factors essential for RSV infection have been reported (**Table 2**), including ATP1A1 (Lingemann et al., 2019), actin-related protein 2 (ARP2) (Mehedi et al., 2016), toll-like receptor 4 (TLR4) and CD14 (Kurt-Jones et al., 2000), ATP binding cassette E1 (ABCE1) (Anderson et al., 2019), Rab5a (Mo et al., 2021), c-Jun N-terminal kinases (JNKs) (Caly et al., 2017), importin β1 (Ghildyal et al., 2005), actin-binding protein cofilin 1(Cof1), caveolae protein Caveolin 2 (Cav2), zinc finger protein ZNF502 (Kipper et al., 2015), Crm1 (Ghildyal et al., 2005), and p32 (Hu et al., 2017).

In this review, we discussed recent advances in RSV receptors and host factors and tried to elucidate their roles in the pathogenesis of RSV infection. A better understanding of the interaction between RSV and the host could help to identify new therapeutic targets and guide future interventions.

## Respiratory Syncytial Virus Receptors

### CX3C Chemokine Receptor 1

CX3CR1 is a seven-transmembrane G-coupled chemokine receptor expressed in various cell types, including NK cells, microglia, macrophages, monocytes, neurons, epithelium, and endothelium (Imai et al., 1997; Kim et al., 2011). In the lung, it is exclusively expressed on motile cilia of lung ciliated epithelial cells (Jeong et al., 2015; Anderson et al., 2020). The fractalkine (CX3CL1) and CX3C motif of the RSV G glycoprotein are only two known ligands for CX3CR1 (Tripp et al., 2001; Kim et al., 2011). The CX3C motifs (aa 169–191), a structure within the cystine noose of G glycoprotein, shares 42% homology with the chemokine domain of fractalkine (Tripp et al., 2001). The interaction between CX3CR1 and fractalkine mediates leukocyte adhesion, activation, and trafficking (Harcourt et al., 2006). The CX3CR1–CX3CL1 axis has been shown to play an important role in the pathogenesis of brain and neurodegenerative diseases, such as Alzheimer’s disease (Lee et al., 2018), but its role in lung diseases, especially in infectious respiratory diseases, is poorly characterized. In RSV infection, the conserved CXC3 motif of its G glycoprotein was able to bind to CX3CR1 *in vitro* and *in vivo* to initiate infection (Jeong et al., 2015; Anderson et al., 2020; Green et al., 2021). Given its expression pattern in the lung, which matched the tropism of RSV infection, studies have shown that CX3CR1 is a more attractive candidate than HSPGs *in vivo*, since HSPGs are a primary receptor on immortalized cell lines but are expressed at much lower levels in the human airway epithelium, the primary site of RSV infection (Anderson et al., 1988; Johnson et al., 2015; Anderson et al., 2020; Green et al., 2021).

The interaction between RSV and CX3CR1 has a crucial role in RSV pathogenesis. It can induce the secretion of multiple cytokines and chemokines secretion, including IL-8, MIG, and fractalkine (Chirkova et al., 2015). In fatal RSV bronchiolitis, the lung tissue was prominent for B-cell infiltration (Reed et al., 2009). Zhivaki et al. showed that RSV could activate neonatal B regulatory (nBreg) cells *via* the interaction between the F protein and BCR, which could subsequently upregulate CX3CR1 expression on nBreg cells. G protein induced RSV attachment and entry by binding to CX3CR1 and mediated IL-10 secretion, inhibiting Th1 cell polarization and resulting in heavy viral load and severe bronchiolitis (Zhivaki et al., 2017). Harcourt et al. indicated that the G protein CX3C motif could reduce the antiviral T-cell response by inhibiting IFN-γ-secreting CX3CR1^+^ T-cell migration to the lung. However, their study suggested that CX3CR1^+^ cytotoxic cells were preferentially recruited to the lung during RSV infection and constituted a major cytotoxic population in the lung tissue (Harcourt et al., 2006). Chirkova et al. suggested that the CX3C motif could impair innate and adaptive immune responses and suppress antiviral activity. The CX3C chemokine motif suppressed IFN-type I/III production and decreased maturation and percentage of IFN-α and tumor necrosis factor alpha (TNF-α)-producing plasmacytoid dendritic cells and TNF-α-producing monocytes (Chirkova et al., 2013). CX3CR1 is highly expressed on Th1 cells, and the fractalkine–CX3CR1 interaction can mediate an amplification circuit of Th1-polarized immune responses (Fraticelli et al., 2001). Mutation of CX3CR1, which decreased the affinity of CX3CR1 to fractalkine, resulted in poor clinical outcomes (Amanatidou et al., 2006). Thus, the CX3C motif might compete with fractalkine for CX3CR1 binding (Chirkova et al., 2013) and subsequently disrupt the fractalkine-induced Th1-polarized response, which is beneficial for the body to rule out viral infection. In addition, binding of the CX3C motif to CX3CR1 led to a significant decrease in the expression of cilium-related genes, such as CC2D2A and CFAP221. The counts of ciliated cells, on average, were reduced significantly in epithelial cell cultures compared to non-infected cultures (Anderson et al., 2021). The binding of RSV G protein to CX3CR1 could also trigger the expression of nucleolin and increase the internalization of RSV, although the specifics in the signaling cascade remained poorly delineated.

The CX3C motif on the G protein is a promising target for prophylactic and therapeutic treatment development. Mutation of the CX3C motif to CX4C could restore the Th1 polarization response (Chirkova et al., 2013). The anti-G mAbs, 131–2G, 3G12, and 3D3, whose binding sites are adjacent to the CX3C motif, could reduce lung viral titers and inflammation in established RSV infection (Caidi et al., 2018).

### Nucleolin and Insulin-Like Growth Factor-1 Receptor

Nucleolin is a protein expressed abundantly in the nucleolus and is also found in different eukaryotic cell compartments, including nucleoplasm, cytoplasm, and cell membrane (Ginisty et al., 1999; Jia et al., 2017). Studies have shown that nucleolin (NCL) is expressed on the apical surface of the human respiratory epithelium (Tayyari et al., 2011; Griffiths et al., 2020). NCL is composed of three domains: the N-terminal domain, which is rich in glutamic acid and aspartic acid and functions in division control; the central domain, also called RNA-binding domains (RBDs) which interact with RNA; and the C- terminal domain, which interacts with nucleic acids and facilities RBD binding (Ginisty et al., 1999; Jia et al., 2017). The shuttling property of NCL could mediate internalization of its binding partners (Koutsioumpa and Papadimitriou, 2014) and thus play a role in virus entry. Studies have been shown that cell surface nucleolin plays a vital role in viral infection, including herpes simplex virus type 1 (Calle et al., 2008), rabies virus (Oksayan et al., 2015), influenza virus (Kumar et al., 2016), respiratory syncytial virus (Tayyari et al., 2011), and some other viruses. During RSV infection, the RBD 1,2 of NCL serves as a receptor for the F protein of RSV (Mastrangelo et al., 2021) and mediates RSV internalization. Although nucleolin is expressed at low levels on the cell surface (Griffiths et al., 2020), it has fairly efficient kinetics of turnover with a half-life <1 h (Hegele, 2012), and the constant turnover of cell surface nucleolin can provide enough NCL for F protein binding.

The IGF1R is a dimeric transmembrane glycoprotein in which the extracellular region is involved in ligand binding and the intracellular region contains a tyrosine kinase domain. The interaction between IGF1R and its natural ligand IGF mainly activates two signaling cascades, the PI3K-AKT/mTOR and mitogen-activated protein kinase (MAPK) pathways (Forbes et al., 2020). The interaction between IGF1R and prefusion RSV F protein could mediate the activation of protein kinase C zeta, facilitating the translocation of nucleolin to the cell membrane (Griffiths et al., 2020) and enhancing RSV internalization.

### Epidermal Growth Factor

EGFR is composed of an extracellular cysteine-rich ligand receptor, a single α-helix transmembrane domain, and an intracellular kinase domain (Ullrich et al., 1984). EGFR signaling has been demonstrated to stimulate macropinocytosis by activating PAK1, leading to changes in membrane and cytoskeletal dynamics (Weerasekara et al., 2019; Lee et al., 2019). EGFR activation and upregulation of its downstream effectors, such as Cdc42 and PAK1, were observed during RSV infection and followed by an increase in extracellular fluid uptake, which indicated RSV-induced micropinocytosis (Krzyzaniak et al., 2013). In addition, the interaction between EGFR and F protein contributes to RSV pathogenesis by promoting fusion and airway mucus secretion (Currier et al., 2016).

### Heparan Sulfate Proteoglycans

HSPGs are found on the basement membranes of most mammalian cell types and in the extracellular matrix. They are responsible for a wide range of functions, such as providing a matrix for cell migration, packing granular contents, protecting cytokines and chemokines from proteolysis, and facilitating cell-extracellular matrix attachment and cell to cell interactions (Sarrazin et al., 2011). HSPGs are utilized by many viruses for attachment and initiation of infection, such as herpesviruses (Flynn and Ryan, 1995), HIV (Mondor et al., 1998), and picornaviruses (Jackson et al., 1996).

RSV G protein contains an HBD adjacent to a conserved cysteine noose, and studies have identified HSPGs as crucial receptors for RSV infection on immortalized cell lines *in vitro* (Krusat and Streckert, 1997; Bourgeois et al., 1998; Feldman et al., 1999; Hallak et al., 2000). The RSV F protein could also interact with HSPGs to facilitate viral attachment and infection (Feldman et al., 2000). Adding heparin-subserted cell lines or reducing the heparan sulfate expression on the cellular surface could significantly reduce RSV infection. However, a lack of HSPGs expression on the apical surface of human bronchial epithelium or basal only expression indicated that HSPG might not be a candidate receptor for RSV to initiate infection *in vivo* (Anderson et al., 2020; Green et al., 2021).

### Intercellular Adhesion Molecule-1

ICAM-1, also known as CD54, is a member of the immunoglobulin superfamily, composed of five consecutively linked extracellular immunoglobulin-like domains (Staunton et al., 1988; Staunton et al., 1990). ICAM-1 is mainly expressed on the surface of immune, endothelial, and epithelial cells and is fairly low, and its expression can be upregulated by cytokines such as IL-1, IFN-γ, and TNF-α (Hubbard and Rothlein, 2000; Yu et al., 2020), in response to inflammatory stimulation. ICAM-1 mediates diverse cellular processes by binding to LFA-1, including leukocyte adhesion, transendothelial migration, signal transduction, and immune response (Bella et al., 1998; Wee et al., 2009; Teijeira et al., 2017). ICAM-1 is a primary receptor for the major groups of rhinoviruses, including all RV-B and most RV-A (Basnet et al., 2019). RSV could also utilize ICAM-1 as a receptor *via* the F protein (Behera et al., 2001). Antibodies targeting ICAM-1 could significantly reduce RSV infection. Studies have demonstrated that RSV infection can significantly upregulate the expression levels of ICAM-1 *in vitro* (Arnold and Konig, 1996; Wang et al., 2000), which are mediated by nuclear factor kappa B (NF-κB), CCAAT/enhancer-binding protein (C/EBP) and interleukin-1α [Patel et al., 1995; Chini et al., 1998]. The interaction between the F protein and ICAM-1 plays a role in RSV pathogenesis. Upregulation of ICAM-1 in RSV-infected respiratory epithelial cells facilitated neutrophil and eosinophil adhesion, which might contribute to airway inflammation, injury, and obstruction (Stark et al., 1996).

## Host Restriction Factors in RSV Infection

### Guanylate Binding Protein 5

Guanylate binding protein 5 (GBP5) belongs to the GBP family, a family of IFN-inducible guanosine triphosphatases (GTPases), which are important for activating innate immunity against a wide variety of intracellular pathogens (Cui et al., 2021). GBP5 is involved in diverse cellular processes, such as inflammasome activation, signal transduction, translation, and exocytosis (Feng et al., 2017). Previous studies have demonstrated that GBP5 can restrict the replication of HIV-1 (Krapp et al., 2016) and influenza A virus (Feng et al., 2017).

GBP5 was identified as a host restriction factor of RSV. It functioned by targeting the SH protein (Li et al., 2020). RSV small hydrophobic (SH) protein is a transmembrane surface glycoprotein located at the Golgi complex and cell surface that functions as cation-selective ion channels in planar lipid bilayers (Gan et al., 2008). GBP5 could interact with SH protein and interfere with SH function by mediating SH oversecretion from cells. The inhibitory property of GBP5 in RSV infection required the localization of GBP5 in the Golgi but not its GTPase activity. In addition, GBP5 could boost the IFN-γ response, which has strong antivirus effect on RSV infection (Li et al., 2020).

### Chemokine Ligand 4

Human CXCL4 is a 70-amino acid protein that is mainly expressed in platelet α-granules and is involved in diverse physiological and pathological processes, such as hematopoiesis, angiogenesis, inflammation, and atherosclerosis (Vandercappellen et al., 2011). CXCL4 has been reported to be a regulator in viral replication and propagation. Studies have indicated that CXCL4 can both inhibit and facilitate HIV infection (Schwartzkopff et al., 2009; Auerbach et al., 2012). CXCL4 was able to inhibit the IFN pathway and enhance DENV replication *in vitro* and *in vivo* (Ojha et al., 2019). CXCL4 had an important role in viral clearance in H1N1-infected mouse models, and CXCL4 knockout mice were complicated with severe lung pathology (Guo et al., 2017).

CXCL4 has a protective effect against RSV infection, and its expression levels are strongly related to disease severity. CXCL4 restricted RSV replication by blocking the binding to HSPGs. CXCL4 could be induced by RSV and attenuate lung inflammation in mouse models. CXCL4 concentration could be an indicator of disease severity. Its concentration in plasma is negatively associated with RSV replication and disease severity due to its inhibitory property in RSV entry and replication, while its concentration in nasopharyngeal aspirates is associated with high viral load and severe infection (Han et al., 2020).

### Extraribosomal L13a

L13a is a ribosomal protein released from the 60S ribosomal subunit (Mazumder et al., 2003). Released L13a could bind the gamma-activated inhibitor of translation (GAIT) elements, a specific RNA hairpin in the 3′ untranslated regions (3′UTR) of the target proinflammatory mRNAs and subsequently cause translational silencing (Sampath et al., 2004; Vyas et al., 2009). Studies had shown its antiviral activity in RSV and severe acute respiratory syndrome coronavirus 2 (SARS-CoV-2) through the VAIT pathway, a pathway similar to GAIT, which silences viral translation by forming an RNA-binding complex targeting the 3′UTR of viral mRNA (Mazumder et al., 2014; Basu et al., 2021). RSV triggered the release of L13a from the ribosome and released L13a-mediated VAIT complex formation, which could bind to the RSV M gene mRNA 3′UTR and silence M protein translation (Mazumder et al., 2014). Since the mRNA silencing property of L13a was observed in RSV-infected non-immune cells, this indicated that the activation of L13a release and formation of the VAIT complex were independent of IFN-γ, which was essential for initiating GAIT. However, the signaling cascade in RSV-infected non-immune cells triggering L13a dissociation remains unclear.

### Interferon-Induced Transmembrane Proteins

IFITM proteins belong to a family of small transmembrane proteins that have been ascribed a role in antiviral activity (Brass et al., 2009; Diamond and Farzan, 2013). They are activated strongly by type I and type II IFNs in the early phase of viral infection and have been demonstrated as a host restriction factor in influenza A virus, HIV, dengue virus, SARS-CoV-2, etc. (Huang et al., 2011; John et al., 2013; Compton et al., 2014; Prelli Bozzo et al., 2021). IFITM has been shown to inhibit RSV infection. IFITM1 located on the cell surface could prevent infection by RSV, and the antiviral activities were related to the cytoplasmic intracellular loop (CIL) domain because mutation of the CIL domain changed its cellular locations significantly, reducing its inhibition of RSV infection. The mice lacking IFITM1 experienced more severe disease after RSV infection, presented by significant weight loss, higher lung viral load, and prominent inflammatory cell infiltration in lungs compared to control groups (Smith et al., 2019). IFITM3 knockout mice showed significant weight loss and cellular infiltration, which indicated that IFITM3 could also restrict RSV infection *in vivo* (Everitt et al., 2013). Zhang et al. showed that IFITM inhibited RSV infection by interfering with viral entry and replication (Zhang et al., 2015). However, the specific mechanism of host–pathogen interactions between IFITM and RSV is less characterized.

### Interferon-Induced Protein 44 and Interferon-Induced Protein 44-Like

IFI44 and IFI44L are both IFN-stimulated genes (ISGs) located on the same chromosome and share 45% amino acid identity. Studies have reported their antiviral activities against HIV, HCV, and influenza A virus (Takahashi et al., 1990; Power et al., 2015; Zhou et al., 2021). In addition, IFI44 and IFI44L had antiproliferative activity (Huang et al., 2018). A bioinformatic screen revealed that IFI44 and IFI44L were upregulated during RSV infection (McDonald et al., 2016; Li et al., 2021). Busse et al. showed that the expression of both genes was upregulated soon after RSV infection *in vitro* and *in vivo* and that they could restrict viral replication and transcription but had no effect on virus attachment (Busse et al., 2020). In addition, the expression levels of IFI44 and IFI44L in infants were negatively related to disease severity.

### Apolipoprotein B mRNA-Editing Enzyme-Catalytic Polypeptide 3G

The APOBEC family is a diverse group containing 11 enzymes involved in humoral immunity, with the functions of RNA and ssDNA editing (Chelico et al., 2009). The deaminase activity of A3G could inhibit the replication of HIV by incorporating into budding virions and inducing hypermutation by deaminating cytidines to uridines (Malim, 2009; Krupp et al., 2013). In addition to its deaminase activity, the property of RNA-binding probably renders its antiviral activity in some virus replication, such as measles, mumps, and respiratory syncytial viruses (Fehrholz et al., 2012; Tiwarekar et al., 2018). The interaction between A3G and RNA impaired viral transcription and inhibited viral replication. Interestingly, A3G enhanced the mutation rate of RSV in a deaminase-independent manner and induced A to G and U to C mutation instead of A3G-specific C to U/T hypermutations (Fehrholz et al., 2012). Overall, A3G could inhibit RSV replication and decrease viral infectivity *in vitro*, but the specific mechanism of inhibition remains unclear.

## Host Factors Essential for RSV Infection

### Toll-Like Receptor 4 and CD14

TLR and CD14 are two essential receptors that contribute to recognizing microbial molecules or products such as lipopolysaccharide (LPS) (Ciesielska et al., 2021), RSV F protein (Kurt-Jones et al., 2000), and chlamydial heat shock protein 60 (Sasu et al., 2001) and boost the innate immune system in the early phase of infection (Takeuchi and Akira, 2010; Wu et al., 2019; Fitzgerald and Kagan, 2020; Ciesielska et al., 2021). RSV F protein interacted with TLR4 and CD14 to stimulate innate response. TLR4 were important to limited viral replication *in vivo*, and RSV infection persists longer in TLR-deficient mice (Kurt-Jones et al., 2000). However, TLR4 might play a role in the pathogenesis of RSV infection. RSV infection could upregulate TLR4 expression on airway epithelial cells and alter the sensitivity of responding to inhaled environmental agents such as LPS, while this response to the LPS exposure was suppressed under normal conditions (Monick et al., 2003). The overexpression of TLR4 and the interaction between TLR4 and LPS induced high secretion of IL-6, which might promote neutrophils survival and infiltration in the lung, increase oxidation activity, and inhibit the Treg suppression function (Detournay et al., 2005; Xie et al., 2009). In addition, studies have demonstrated that polymorphisms of TLR4 and CD14 are related to disease severity (Puthothu et al., 2006; Goutaki et al., 2014; Zhou et al., 2016). TLR4 and CD14 amino acid variants might influence the interaction between RSV F protein and them, which results in differences in the innate immune response.

### ATP1A1

ATP1A1 is the α-subunit of the NA^+^, K^+^-ATPase complex, a protein forming the ion channel responsible for Na^+^ and K^+^ across the plasma membrane. It contains 10 transmembrane helices embedding the ATPase complex in the plasma membrane (Morth et al., 2011; Lingemann et al., 2019). The cytoplasmic tail of ATP1A1 activates the cellular kinase c-Src (Tian et al., 2006) and triggers the phosphorylation of EGFR to induce micropinocytosis (Donepudi and Resh, 2008). Studies have shown that ATP1A1 has a role as a pro-viral factor in various viral infections, such as SARS-CoV-2 (Schmidt et al., 2021), Ebola virus (Garcia-Dorival et al., 2014), hepatitis C virus (Lussignol et al., 2016), and mammarenaviruses (Iwasaki et al., 2018).

ATP1A1 has a positive effect on RSV-induced EGFR-mediated macropinocytosis. ATP1A1 formed clusters during early RSV infection, and the localization of these clusters were adjacent to the F protein. This phenomenon could be reproduced with a UV-inactivated RSV, which indicated that the formation of the ATP1A1 cluster was independent of viral replication and transcription of the complete viral gene. Activated ATP1A1 triggered the EGFR tyrosine 845 phosphorylation *via* the c-Src-kinase pathway and ultimately induced macropinocytosis of RSV. Within the macropinosomes, the RSV F protein underwent a second, highly efficient cleavage to become fusion competent and infectious (Krzyzaniak et al., 2013; Lingemann et al., 2019). However, Lingemann et al. could not identify any binding of RSV protein to ATP1A1. The possible reason might be either the unstable interaction between RSV protein and ATP1A1 or there were as-yet unknown upstream effectors (Lingemann et al., 2019).

### Actin-Related Protein 2

ARP2 is a large subunit of the actin-related protein 2/3 (Arp2/3) complex, which is an important regulator of actin polymerization (Hurst et al., 2004). Actin polymerization mediates morphological changes responsible for diverse cellular processes, including division, phagocytosis, and migration (Swaney and Li, 2016). Actin plays a pivotal role in the RSV life cycle and is involved in the processes of virus transcription, viral morphogenesis, assembly, and budding (Burke et al., 1998; Kallewaard et al., 2005; Jeffree et al., 2007).

ARP2 was identified as a host factor facilitating RSV infection by a genome-wide siRNA screen. Its expression was independent of RSV infection. The ARP2 knockout A549 cell line showed reduced release of progeny RSV particles, indicating that ARP2 might be important for budding and virus production. ARP2 is also involved in the processes of RSV F protein-induced filopodia formation, which facilitates cellular motility and promotes virus spreading to neighboring uninfected cells (Mehedi et al., 2016).

### ATP Binding Cassette E1

ABCE1 is a member of the superfamily of ATP binding cassette proteins. It was originally described as a ribonuclease L inhibitor (Bisbal et al., 1995). ABCE1 is involved in diverse physiological and pathological processes including regulation of mitochondrial mRNA stability, suppression of IFN-induced antiviral activity, promotion of tumor cell proliferation, and antiapoptosis (Lingappa et al., 2006; Tian et al., 2012). Studies have demonstrated its positive role in HIV-1 replication (Zimmerman et al., 2002; Lingappa et al., 2006). ABCE1 was identified as a host factor essential for measles, mumps, and RSV by a genome-scale RNAi screen. It is essential for viral protein synthesis and facilitates the accumulation of viral proteins, which are important for virus replication and progeny virus assembly (Anderson et al., 2019).

### Rab5a

Rab5a belongs to the Rab subfamily of small GTPases that are involved in various cellular processes, such as cell growth, differentiation, intracellular transportation, and signal transduction (Sheng et al., 2014). A previous study showed that Rab5 was required in the formation of macropinosomes and mediated macropinocytosis of RSV (Krzyzaniak et al., 2013). Rab5a might be able to hijack the innate immune response by reducing IFN regulatory factor (IRF) production and subsequently inhibiting the IFN-γ production in RSV infection. The expression of Rab5a was increased in the RSV-infected airway epithelium and Rab5a-deletion-reduced RSV replication and airway inflammation. Moreover, the level of Rab5a was correlated with disease severity in RSV-infected infants (Mo et al., 2021). Altogether, Rab5a might be a host factor essential for RSV infection.

### c-Jun N-Terminal Kinases

JNKs are known as stress-activated proteins that belong to the MAPKs and regulate diverse cellular functions, such as proliferation, apoptosis, and autophagy. Cellular stress induced by viral infection, bacterial toxins, and proinflammatory cytokines strongly activates JNKs (Lee et al., 2016). JNKs were demonstrated to facilitate some viral infections, such as VZV (Zapata et al., 2007), influenza A virus (Zhang et al., 2016), and HCV (Takaki et al., 2017). JNKs were implicated as a pivotal host factor essential for RSV infection (Caly et al., 2017). RSV was able to increase JNK phospho-activation, and activated JNKs could suppress the expression of TNF-α (Stewart et al., 2006), a critical antiviral cytokine against RSV. JNKs were associated with RSV assembly and progeny virion release. Inhibition of JNK1/2 signaling could reduce RSV virion release by trapping virions in the host cell cytoplasm (Caly et al., 2017).

### Host Factors Utilized by RSV M Protein

Matrix (M) protein plays as a key role in RSV life cycles. In early infection, the M protein is imported into the nucleus by importin β1 (Ghildyal et al., 2005) and inhibits host cell transcription (Ghildyal et al., 2002). In late infection, the M protein translocates to the cytoplasm, mainly within inclusion bodies, and promotes RSV replication and release (Ghildyal et al., 2009). Several reported host factors essential for efficient RSV infection have been demonstrated to function by interacting with the RSV M protein. The actin-binding protein cofilin 1 (Cof1), caveolae protein Caveolin 2 (Cav2), and the zinc finger protein ZNF502 were identified as host factors essential for RSV replication by novel microfluidics screening (Kipper et al., 2015). Cof1 was probably trapped within the inclusion body by the M protein and led to an increase in F-actin filaments, which promoted RSV protein trafficking and virus transcription in inclusion bodies. Cav2 enhanced RSV infection by maintaining lipid raft integrity, which is essential for the stability of viral filaments and the release of progeny viruses. Crm1 could mediate M protein shuttling to the cytoplasm. The M protein within cytoplasm inclusion bodies was important for virus assembly (Ghildyal et al., 2009). The mitochondrial protein p32/HAPB1/gC1qR/C1qbp is an important protein in maintaining mitochondrial structures (Hu et al., 2013). RSV could change the distribution of mitochondria in the cytoplasm. p32 and mitochondria adjacent to viral inclusion bodies were required for efficient RSV replication. In addition, knockdown of p32 can significantly reduce RSV production (Hu et al., 2017).

## Conclusion

RSV remains a major cause of lower respiratory tract infections and mortality in children and the elderly. Mainstream therapies remain limited to supportive care. We still do not have a licensed vaccine available to date. The use of prophylaxis and the therapeutic RSV-specific antibody palivizumab is restricted to a minor fraction of the population at high risk due to its uncertainty of clinical values and high costs. The development of vaccines and RSV-specific therapies has been hampered by poor characterization of RSV pathogenesis both in virus entry and immune responses to viruses. Several receptors of RSV have been identified, and antibodies targeting them could significantly reduce RSV replication. Among these receptors, CX3CR1 is the most promising candidate either for delineating the tropism of RSV or taking as a potential therapeutic target because of its expression pattern in the human airway epithelium. In addition to receptors, host factors play an important role in the virus–host interaction. Host restriction factors can inhibit virus replication by interfering with almost every step of the viral life cycle, while there is another group of host factors that are essential for virus infection. The identification of RSV receptors and host factors helps us to identify potential prophylaxis and therapeutic targets and offers opportunities for vaccine and medicine development against RSV infection.

## Author Contributions

This work was a collaboration among all of the authors. ZF wrote the initial draft of the manuscript. ZX reviewed the manuscript. LX proposed the original idea and reviewed the manuscript. All authors contributed to the article and approved the submitted version.

## Funding

This work was supported by grants from the National Major S & T Research Projects for the Control and Prevention of Major Infections Diseases in China (2017ZX10103004-004 and 2018ZX10305409-001-004), CAMS Innovation Fund for Medical Sciences (CIFMS, 2019-12M-5-026), and Respiratory Research Project of National Clinical Research Center for Respiratory Diseases (HXZX-202106).

## Conflict of Interest

The authors declare that the research was conducted in the absence of any commercial or financial relationships that could be construed as a potential conflict of interest.

## Publisher’s Note

All claims expressed in this article are solely those of the authors and do not necessarily represent those of their affiliated organizations, or those of the publisher, the editors and the reviewers. Any product that may be evaluated in this article, or claim that may be made by its manufacturer, is not guaranteed or endorsed by the publisher.
